# Muscle Synergies During Walking in Children With Cerebral Palsy: A Systematic Review

**DOI:** 10.3389/fphys.2020.00632

**Published:** 2020-07-02

**Authors:** Annike Bekius, Margit M. Bach, Marjolein M. van der Krogt, Ralph de Vries, Annemieke I. Buizer, Nadia Dominici

**Affiliations:** ^1^Department of Human Movement Sciences, Amsterdam Movement Sciences, Institute of Brain and Behavior Amsterdam, Vrije Universiteit Amsterdam, Amsterdam, Netherlands; ^2^Department of Rehabilitation Medicine, Amsterdam Movement Sciences, Amsterdam UMC, Vrije Universiteit Amsterdam, Amsterdam, Netherlands; ^3^Medical Library, Vrije Universiteit Amsterdam, Amsterdam, Netherlands

**Keywords:** muscle synergy, cerebral palsy, typically developing, children, gait

## Abstract

**Background:** Walking problems in children with cerebral palsy (CP) can in part be explained by limited selective motor control. Muscle synergy analysis is increasingly used to quantify altered neuromuscular control during walking. The early brain injury in children with CP may lead to a different development of muscle synergies compared to typically developing (TD) children, which might characterize the abnormal walking patterns.

**Objective:** The overarching aim of this review is to give an overview of the existing studies investigating muscle synergies during walking in children with CP compared to TD children. The main focus is on how muscle synergies differ between children with CP and TD children, and we examine the potential of muscle synergies as a measure to quantify and predict treatment outcomes.

**Methods:** Bibliographic databases were searched by two independent reviewers up to 22 April 2019. Studies were included if the focus was on muscle synergies of the lower limbs during walking, obtained by a matrix factorization algorithm, in children with CP.

**Results:** The majority (*n* = 12) of the 16 included studies found that children with CP recruited fewer muscle synergies during walking compared to TD children, and several studies (*n* = 8) showed that either the spatial or temporal structure of the muscle synergies differed between children with CP and TD children. Variability within and between subjects was larger in children with CP than in TD children, especially in more involved children. Muscle synergy characteristics before treatments to improve walking function could predict treatment outcomes (*n* = 3). Only minimal changes in synergies were found after treatment.

**Conclusions:** The findings in this systematic review support the idea that children with CP use a simpler motor control strategy compared to TD children. The use of muscle synergy analysis as a clinical tool to quantify altered neuromuscular control and predict clinical outcomes seems promising. Further investigation on this topic is necessary, and the use of muscle synergies as a target for development of novel therapies in children with CP could be explored.

## Introduction

Walking is the most common form of locomotion adopted by humans and limbed animals, and it requires the activation of numerous muscles. It has been theorized, that in order to coordinate this complex behavior, the central nervous system controls basic building blocks, referred to as muscle synergies or motor modules, rather than individual muscles. Muscle synergies are defined as temporal basic activation patterns of different groups of muscles with a corresponding weighting coefficient for every muscle. Each synergy contains multiple muscles and every muscle can contribute to multiple synergies (Ivanenko et al., 2005; Hart and Giszter, 2010; Dominici et al., 2011; Bizzi and Cheung, 2013).

Over the past years, researchers applied muscle synergies as a framework to analyze neuromuscular control in both healthy subjects and individuals with neurological disorders. Generally, muscle synergies are extracted from electromyography (EMG) using matrix factorization algorithms, like the non-negative matrix factorization (NMF), independent component analysis, or factor analysis (Lee and Seung, 1999). In the healthy population, four or five synergies extracted from a large number of EMGs are required during normal walking and these synergies also account for stride-to-stride variability and various speeds (Ivanenko et al., 2004; Cappellini et al., 2006; Clark et al., 2010; Tang et al., 2015). The muscle synergies of healthy adults (“mature synergies”) are often used as a template to compare the results of synergy analyses of pathological gait. Muscle synergies appear to be altered in the adult population after brain injury. It has been shown that the number of muscle synergies in post-stroke individuals during walking is reduced compared to unimpaired individuals due to merging of the “mature synergies” observed in healthy adults (Clark et al., 2010). These findings correlate with the degree of motor impairment which might reflect a simplified control strategy of the central nervous system in moderate to severely impaired post-stroke individuals. However, it is unclear whether and how this change in synergy organization can be generalized to other clinical populations and how it relates to gait abnormality.

Cerebral palsy (CP) is a neurodevelopmental motor disorder caused by non-progressive lesions in an immature brain (Himmelmann and Uvebrant, 2018). CP has a wide clinical spectrum, with mobility varying from walking without aids, to being completely wheelchair dependent. The Gross Motor Function Classification System (GMFCS) is used to classify functional mobility in CP. Various diagnostic subtypes exist, based on motor type and distribution of CP, that is, spastic, dyskinetic, or ataxic, and unilateral or bilateral CP, respectively (Bax et al., 2005). Individuals with CP who learn to walk, do so after their brain injury, in contrast to adult stroke survivors who have years of walking experience prior to the brain lesion. In typically developing (TD) children, the number of basic muscle activation patterns increases from two in stepping neonates to four in toddlers, just after their first independent steps (Dominici et al., 2011). The early brain injury in children with CP may lead to a different development of muscle synergies, which might be an underlying factor of abnormal walking patterns. Studies on muscle synergies in children with CP are scarcer than in stroke and have used diverse methods to calculate synergies. Methodological choices in factorization methods, filtering conditions, the number of muscles recorded, and the recording quality appear to influence the outcomes of the synergy calculations (Steele et al., 2013; Santuz et al., 2017; Shuman et al., 2017).

Several types of treatment exist to improve gait quality and functional mobility in children with CP. Recent research has identified the possibility that muscle synergies can predict effectiveness of therapies in children with CP (Schwartz et al., 2016; Shuman et al., 2016, 2018; Oudenhoven et al., 2019). A better insight into the neuromuscular control mechanisms underlying the altered muscle activation patterns in children with CP could possibly help to improve therapy choices and functional mobility outcomes. In addition, more knowledge about these mechanisms can be important for the interpretation of clinical signs of CP at an early age, improve indication for therapy in individual patients, and might even be used to develop new diagnostic tools (Cheung et al., 2012).

The present systematic review aims to give an overview of the existing studies investigating muscle synergies in children with CP during walking to evaluate the current knowledge on this topic. The primary aim is to examine how muscle synergies in children with CP differ from those exhibited by TD children during walking by investigating the quantification and structure of synergies, and the variability of synergies between and within children with CP. Second, we aim to examine the predictability of treatment outcomes using muscle synergy characteristics, and the effect of treatment on muscle synergies in children with CP.

## Methods

A systematic review protocol was developed based on the Preferred Reporting Items for Systematic Reviews and Meta-Analyses (PRISMA)-statement (www.prisma-statement.org). It is registered on PROSPERO and can be accessed online (number: CRD42019149109).

### Search Strategy

A comprehensive search was done in the bibliographic databases PubMed, Embase.com, and Web of Science (Core collection), in collaboration with a medical librarian (RV). Databases were searched up to 22 April 2019. The following terms were used including synonyms and closely related words (see Supplementary Information) as index terms or free-text words: “Muscle synergy,” “Cerebral palsy,” “Typically developing,” “Children,” “Walking”. The search was performed without date, language, or publication status restriction. The full search strategies for all databases can be found in the online Supplementary Information (see Appendix).

### Study Selection

Studies were eligible for inclusion if they met the following inclusion criteria: (1) children with CP younger than 19 years old, and in case of mixed populations: the majority of the investigated population younger than 19, (2) the focus of the study was on muscle synergies of the lower limb during walking, (3) use of a matrix factorization algorithm to obtain the muscle synergies. Studies were excluded if, (a) it was a conference abstract, (b) it was a conference paper, but a full paper was published afterwards, (c) the study focused on muscle synergies of the upper limb, and (d) the article was a review or protocol.

After exclusion of duplicate articles, two independent reviewers (AB and MB) performed a title and abstract screening on the residual articles. Thereafter, the reviewers assessed the eligibility of the remaining articles in a full-text screening. Any in- and exclusion conflict among the reviewers was discussed until a consensus was reached. Study designs were defined as being either a cross-sectional, case-control, or retrospective cohort study. Methodological quality and risk of bias of the included articles was assessed using the Downs and Black checklist by the same two independent reviewers (Downs and Black, 1998). In the original scale it is possible to score up to 32 points, but we used a modified version that was applicable for the types of studies included in this systematic review, as has been done in other reviews using the Downs and Black scale (Gorber et al., 2007; Hebert-Losier et al., 2009). This left a maximum total score of 14 points for cross-sectional studies, and 15 points for case-control and cohort studies. Each study was assigned a grade of “excellent” (13–15 points), “good” (10–12 points), “fair” (7–9 points) or “poor” (<7 points). Any disagreements in grading among the reviewers was discussed until consensus was reached. Articles were not excluded based on poor quality, but this played a role in the overall assessment of the article in the review.

### Data Extraction and Analysis

Data extraction of the included articles was performed independently by AB and MB. Subject characteristics (age, CP type and distribution, GMFCS), study methods (number of strides analyzed, number of muscles recorded, EMG pre-processing steps, analysis criteria), and outcome measures (muscle synergies) were summarized in a table. The main outcome measures analyzed in this review were: (1) quantification of muscle synergies during walking, such as total number of synergies, VAF_1_ and walk-DMC, and (2) the spatial and temporal structure of muscle synergies during walking. These outcome measures were assessed in both children with CP and TD children, and pre- and post-treatment in some studies. In addition, variability in number and structure of synergies between and within subjects in the group of children with CP was evaluated.

## Results

### Study Selection

**Figure 1:**
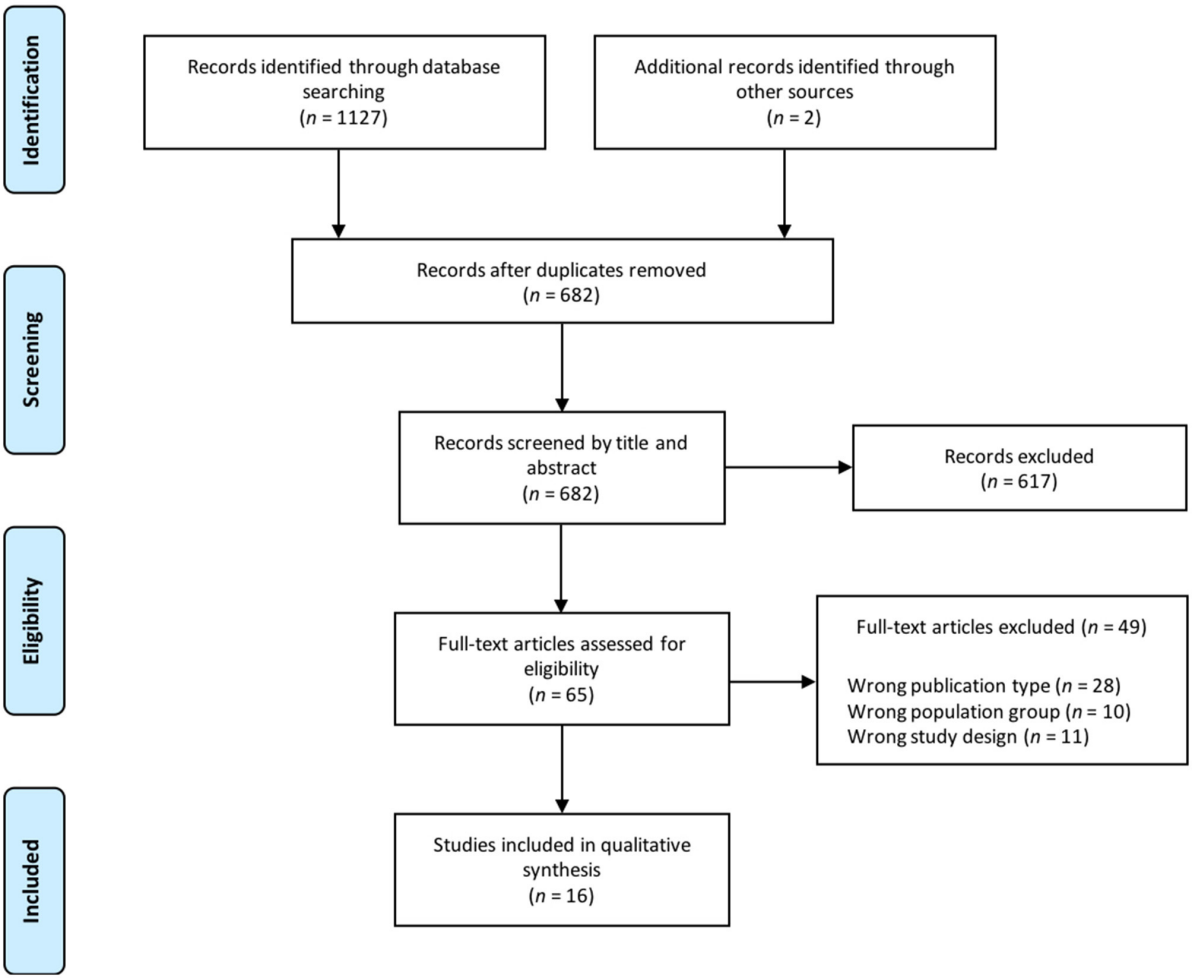
Flow chart article selection.

The electronic search in the cited databases and manual searching of reference lists identified 1127 articles, plus 2 references via additional sources (Figure 1). After duplicate removal, 682 articles were screened on title and abstract, from which 617 were excluded, mostly because of differing target populations (e.g., animals, other diagnosis, or age) or study design (i.e., no muscle synergy analysis during walking involved). Full-text screening of 65 articles left a total of 16 articles that were selected for this review, reasons for exclusion of 49 articles are noted in Figure 1.

### Study Characteristics

Twelve of the 16 articles compared children with CP with TD children, four included only children with CP. The studies varied in sample size, from 3 to 549 children with CP and 8 to 84 TD children. All studies included children with age ranged from 1 to 16 years, in only one study (Steele et al., 2015) older individuals with CP were also included. All studies included children with spastic CP, except for one that included one dyskinetic child (Tang et al., 2015), and GMFCS levels varied from I to IV. An overview of all studies is given in Table 1.

**Table 1:** Summary of the selected study characteristics.

**Study**	**Subjects**	**Age (yrs)**	**CP type & distribution**	**GMFCS**	**N strides analyzed**	**N muscles (= total)**	**EMG pre-processing**	**Analysis** **criteria**	**Synergies**			
									**Total N**	**VAF_**1**_ (%)**	**Walk-DMC**	**Structure**
Cappellini et al. (2016)	CP: 35 TD: 33	CP: 2.3–11.8 TD: 1.0–11.8	Spastic (16 uni, 19 bi)	I, II, III	CP: 50 ± 24 TD: 72 ± 28	11 bi (= 22)	HP: 30 Hz, Demeaned LP: 10 Hz	RMSE of VAF vs. n curve <10^−4^	CP: 4 TD: 4	-	-	Temporal: CP ≠ TD
Cappellini et al. (2018)	CP: 14 TD: 14	CP: 3.0–11.1 TD: 3.3–11.8	Spastic (5 uni, 9 bi)	I, II	CP: 35 ± 5 TD: 27 ± 3	11 bi (= 22)	HP: 30 Hz, LP: 10 Hz	RMSE of VAF vs. n curve <10^−4^	CP: 4 TD: 4	-	-	Temporal: CP ≠ TD
Hashiguchi et al. (2018)	CP: 13 TD: 10	CP: 12.8 ± 3.8 TD: 13.4 ± 0.5	NG	I, II, III	5	8 uni (= 8)	BP: 20–250 Hz, LP: 10 Hz	VAF>90%	CP: 55% = 2, 30% = 3, 15% = 4 TD: 10% = 3, 60% = 4, 30% = 5	-	-	-
Tang et al. (2015)	CP: 12 TD: 8 AD: 10	CP: 5.8 (3.7–9.0) TD: 6.1 (4.5–9.2) AD: 24.5 (23–26)	Spastic (2 uni, 9 bi) 1 Dysk	I, II, III, IV	>20	8 bi (= 16)	HP: 50 Hz, Demeaned, LP: 10 Hz	VAF>95%	CP: 37.5% = 2^*^, 29.2% = 3^*^, 33.3% = 4^*^ TD: 31.2% = 3, 68.8% = 4 AD: 100% = 4	-	-	CP ≠ TD&AD SCA: CP = 57.0 ± 16.8, TD = 84.2 ± 11.8, AD = 95.7 ± 2.0
Yu et al. (2019)	CP: 18 TD: 8	CP: 4.4 (2.3–6.5) TD: 4.4 ± 1.4	Spastic (bi)	I, II, III	8 (NMF on each stride separately)	8 bi (= 16)	HP: 50 Hz, Demeaned, LP: 10 Hz	VAF_4_	CP: GMFCS I/II = 4, GMFCS III = 3 TD: 4	-	-	Spatial: CMFCS I/II = TD GMFCS III ≠ TD Temporal: CP ≠ TD
Torricelli et al. (2014)	CP: 3	15, 14, 14	Spastic (bi)	II	>3	8 bi (= 16)	BP: 20–400 Hz, Demeaned, LP: 5 Hz	VAF>90%	2	-	-	CP ≠ AD
Shuman et al. (2017)	CP: 113 TD: 73	CP: I: 10.4 ± 4.8, II: 10.9 ± 5.8, III: 12.2 ± 9.4 TD: 10.3 ± 3.5	Spastic (bi)	I, II, III	NG	5 bi (= 10)	HP: 40 Hz, LP: 4, 6, 8, 10, 20, 30, 40 Hz	VAF>90% VAF_1_ Walk-DMC Different LP cut-offs	**LP 4 Hz** CP: 2.1 ± 0.6 TD: 2.9 ± 0.4 **LP 40 Hz** CP: 2.9 ± 0.4 TD: 3.4 ± 0.5	**LP 4 Hz** CP: I = 80^†^, II = 84^†^, III = 88^†^ TD: 72 **LP 40 Hz** CP: I = 75^†^, II = 78^†^, III = 82^†^ TD: 62.4	**LP 4 Hz** CP: I = 82^†^, II = 75^†^, III = 64^†^ TD: 100 **LP 40 Hz** CP: I = 84^†^, II = 77^†^, III = 67^†^ TD: 100	-
Steele et al. (2019)	CP: 20	10.4 (6.2–13.6)	Spastic (bi)	I, II, III	>3	5 bi (= 10)	HP: 25 Hz, LP:10 Hz	VAF>95% VAF_1_	3.1 (range 2–4)	81.4 ± 5.5	-	-
Shuman et al. (2019)	CP: 147 TD: 31	CP: BoNT-A: 6.8 ± 2.9, SDR: 9.3 ± 2.0, SEMLS: 12.1 ± 3.1 TD: 9.3 ± 2.8	Spastic (33 uni, 144 bi)	I, II, III	NG	8 bi (= 16)	HP: 20 Hz, LP: 10 Hz	VAF>90% VAF_1_ Walk-DMC	CP: 2.8 ± 0.6 TD: 4.2 ± 0.4	CP (pre-treatment): BoNT-A: 79.1 ± 6.2, SDR: 80.1 ± 4.9, SEMLS: 80.2 ± 5.9 TD: 64.4 ± 3.1	Improved post-treatment	Spatial & temporal: Pre-treatment CP ≈ TD
Oudenhoven et al. (2019)	CP: 36	7.2 (4–13)	Spastic (bi)	I, II, III	3	5 bi (= 10)	HP: 20 Hz, LP: 2 Hz	VAF>90%	Higher N = better treatment outcomes	No correlation with treatment outcomes	-	-
Kim Y. et al. (2018)	CP: 20 TD: 8	CP: 12.5 ± 3.3 TD: 12.0 ± 2.6	Spastic (17 uni, 3 bi)	I, II	5 (NMF on each stride separately)	8 bi (= 16)	HP: 35 Hz, LP: 5 Hz	VAF>90% VAF_1_ Walk-DMC	Mean per stride CP: 3.4 ± 0.3 TD: 3.8 ± 0.2	CP: 71 ± 4 TD: 61 ± 3	CP: 65 ± 14.2 (40.2–91.3) TD: 100 ± 10 (85.1–113.0)	Spatial: CP = TD Temporal: CP ≠ TD
Steele et al. (2015)	CP: 549 TD: 84	CP: 9.8 (7.4–13.3)^#^ TD: 10.3 (7.6–13.0)^#^	Spastic (122 uni, 427 bi)	I, II, III, IV	1	5 bi (= 10)	BP: 20–400 Hz, LP: 10 Hz	VAF>90%	CP: >80% = 1 or 2 TD: >60% = 3	CP: 84.2 (83.7–84.7) TD: 74.6 (71.3–76.1)	CP: 86.2 (85.5–86.9) TD: 100 (97.9–102.1)	Spatial: CP = TD Temporal: CP ≠ TD
Shuman et al. (2016)	CP: 5 TD: 6	CP: 10.2 (6.0–13.0) TD: 10.3 (6.0–13.0)	Spastic (2 uni, 3 bi)	I	CP: 47.5 ± 19.6 (24–81) TD: 44.8 ± 15.9 (25–78)	8 bi (= 16)	HP: 40 Hz, LP: 4 Hz	VAF_1_	-	CP: 77.2 ± 4.1 TD: 68.4 ± 2.3	-	-
Goudriaan et al. (2018)	CP: 15 TD: 15 DMD: 15	CP: 8.9 (7.6–9.8)^#^ TD: 8.6 (7.3–10.0)^#^ DMD: 8.7 (6.8–9.9)^#^	Spastic (8 uni, 7 bi)	I, II	10	8 bi (= 16)	BP: 20–450 Hz, LP: 10 Hz	VAF_1_	-	CP: 74 TD: 65 DMD: 60	-	-
Schwartz et al. (2016)	CP: 473	7.7 ± 3.3	NG	I, II, III	>4	8 bi (= 16)	NG	Walk-DMC	-	-	Higher walk-DMC pre- treatment = better outcomes	-
Shuman et al. (2018)	**Centre 1** CP: 473 TD: 84 **Centre 2** CP: 163 TD: 12	**Centre 1** CP: 7.5 ± 3.4 **Centre 2** CP: 9.3 ± 2.7	NG	I, II, III	NG	**Centre 1** 8 bi (= 16) **Centre 2** 4 bi (= 8)	HP: 20 Hz, LP: 10Hz	Walk-DMC	-	-	CP < TD Higher walk-DMC pre- treatment = better outcomes	-

CP, cerebral palsy; TD, typically developing; AD, adults; DMD, duchenne muscular dystrophy; Uni, unilateral; Bi, bilateral; Dysk, dyskinetic; GMFCS, gross motor function classification system; N, number of synergies; HP, high-pass filter, LP, low-pass filter; NMF, non-negative matrix factorization; VAF, variance accounted for; VAF_1_, variance accounted for by one synergy (%); RMSE, root mean square error; Walk-DMC, dynamic motor control index during walking; SCA, synergy comprehensive assessment; NG, not given; BoNT-A, Botulinum Toxin Type A; SDR, Selective Dorsal Rhizotomy; SEMLS, Single-Event Multilevel Surgery. Age and number of strides are given as mean (± 1 standard deviation or the range when provided by the authors) unless marked by a #, as this signifies the median (25th-75th percentile).

* Values in figure and text are not in agreement, so these values are extracted from the figure;

† *Signifies that values are extracted from graphical representations and are not precise*.

### Risk of Bias

Results of the methodological quality assessment are presented in Table 2. Eight studies used a cross-sectional design (Torricelli et al., 2014; Tang et al., 2015; Cappellini et al., 2016, 2018; Goudriaan et al., 2018; Hashiguchi et al., 2018; Kim Y. et al., 2018; Yu et al., 2019), four used case-control designs (Steele et al., 2015, 2019; Shuman et al., 2016, 2017), and four were retrospective cohort studies (Schwartz et al., 2016; Shuman et al., 2018, 2019; Oudenhoven et al., 2019). Quality scores ranged from 5 to 13, one study received the grade “poor” (Torricelli et al., 2014), ten studies “fair” (Tang et al., 2015; Cappellini et al., 2016, 2018; Shuman et al., 2016, 2018, 2019; Hashiguchi et al., 2018; Kim Y. et al., 2018; Steele et al., 2019; Yu et al., 2019), three studies “good” (Steele et al., 2015; Shuman et al., 2017; Goudriaan et al., 2018), and two studies “excellent” (Schwartz et al., 2016; Oudenhoven et al., 2019).

**Table 2:** Results of methodological quality assessment.

**Study**	**Study design**	***Reporting***										***Ext. validity***			***Int. validity - bias***							***Int. validity—confounding***						***Power***	**Total** **score**	**Grade**
		**1**	**2**	**3**	**4**	**5**	**6**	**7**	**8**	**9**	**10**	**11**	**12**	**13**	**14**	**15**	**16**	**17**	**18**	**19**	**20**	**21**	**22**	**23**	**24**	**25**	**26**	**27**		
Cappellini et al. (2016)	Cross-sectional	1	1	1			1	1			0	0*	0*				1		1		1	1	0*					0	9	Fair
Cappellini et al. (2018)	Cross-sectional	1	1	1			1	1			0	0*	0*				1		1		1	1	0*					0	9	Fair
Goudriaan et al. (2018)	Cross-sectional	1	1	1			1	1			0	0*	0*				1		1		1	1	0*					1	10	Good
Hashiguchi et al. (2018)	Cross-sectional	1	1	1			1	1			0	0*	0*				1		1		1	1	0*					0	9	Fair
Kim Y. et al. (2018)	Cross-sectional	1	1	1			1	1			1	0*	0*				1		1		1	0*	0*					0	9	Fair
Tang et al. (2015)	Cross-sectional	1	1	1			1	1			0	0*	0*				1		1		1	0*	0*					0	8	Fair
Torricelli et al. (2014)	Cross-sectional	1	1	0			1	0			0	0	0				1		0		1	0*	0*					0	5	Poor
Yu et al. (2019)	Cross-sectional	1	1	1			1	1			1	0*	0*				1		1		1	0*	0*					0	9	Fair
Shuman et al. (2016)	Case-control	1	1	0			1	1			1	0*	0*				1	0	1		1	0*	0*					0	8	Fair
Shuman et al. (2017)	Case-control	1	1	1			1	1			0	0*	1				1	0*	1		1	1	0*					0	10	Good
Steele et al. (2015)	Case-control	1	1	1			1	1			0	1	1				1	0*	1		1	1	1					0	12	Good
Steele et al. (2019)	Case-control	1	1	0			1	1			0	0*	0*				1	1	1		1	1	0*					0	9	Fair
Schwartz et al. (2016)	Retrospective cohort	1	1	1			1	1			0	1	1				1	1	1		1	1	1					0	13	Excellent
Shuman et al. (2018)	Retrospective cohort	1	1	1			1	1			0	0	0*				1	0*	1		1	1	0*					0	9	Fair
Shuman et al. (2019)	Retrospective cohort	1	1	0			1	1			1	0*	0*				1	0*	1		1	1	0*					0	9	Fair
Oudenhoven et al. (2019)	Retrospective cohort	1	1	1			1	1			0	1	1				1	1	1		1	1	1					0	13	Excellent

1: yes; 0:no; 0*: unable to determine; Ext.: external; Int.: internal; maximum total scores: cross-sectional = 14; case-control and cohort = 15.

### Calculation of Synergies

All studies used NMF to obtain the muscle synergies from the original (processed) muscle activity. Muscle activity was recorded during overground walking using surface EMG in all cases, 4 to 11 muscles were included per leg, as specified in Table 3. The raw EMG data was most commonly processed using the following steps: high-pass filtered, demeaned (optional), rectified, low-pass filtered, amplitude scaled, and time-normalized. NMF has non-negative constraints, meaning that the original EMG data cannot be negative, and the most used algorithm is the “multiplicative update rule” algorithm presented by Lee and Seung (1999).

**Table 3:** Overview of the recorded and analyzed muscles.

**References**	**Muscles**	**Recorded sides**	**Analyzed sides**	**N analyzed muscles in NMF**
Cappellini et al. (2016)	TA, SOL, GL, GM, RF, VM, VL, ST, BF, TFL, GLMax	Bi	Both legs separately	11 for each leg
Cappellini et al. (2018)	TA, SOL, GL, GM, RF, VM, VL, ST, BF, TFL, GLMax	Bi	Both legs separately	11 for each leg
Hashiguchi et al. (2018)	TA, SOL, GL, RF, VM, ST, BF, GLMed	Uni	Most affected leg	8
Tang et al. (2015)	TA, SOL, GL, VL, RF, ST, BF, TFL	Bi	Both legs separately	8 for each leg
Yu et al. (2019)	TA, SOL, GL, VL, RF, ST, BF TFL	Bi	Both legs separately	8 for each leg
Torricelli et al. (2014)	TA, GM, VM, VL, RF, AL, ST, BF	Bi	Both legs separately	8 for each leg
Shuman et al. (2017)	TA, GM, RF, ST, BF	Bi	Random leg	5
Steele et al. (2019)	TA, GM, VL, RF, ST	Bi	Both legs separately	5 for each leg
Shuman et al. (2019)	TA, SOL, GM, RF, VL, ST, BF, GLMed	Bi	Most affected or random leg	8
Oudenhoven et al. (2019)	TA, GM, RF, VL, ST	Bi	Most affected leg	5
Kim Y. et al. (2018)	TA, GM, RF, ST	Bi	Both legs combined	8
Steele et al. (2015)	TA, GM, RF, ST, BF	Bi	Uni CP: most affected leg Other individuals: random leg	5
Shuman et al. (2016)	TA, SOL, GL, RF, VL, ST, BF, GLMed	Bi	Uni CP: most affected leg TD & Bi CP: both legs	8 or 16
Goudriaan et al. (2018)	TA, GM, RF, ST, GLMed	Bi	Most affected or involved leg	5
Schwartz et al. (2016)	TA, GM, RF, ST	Bi	NG	8
Shuman et al. (2018)	**Centre 1:** TA, GM, RF, ST **Centre 2:** TA, SOL, GM, RF, VL, ST, BF, GLMed	Bi	Most affected or random leg	**Centre 1:** 4 **Centre 2:** 8

*N, number; TA, tibialis anterior; SOL, soleus; GM, gastrocnemius medialis; GL, gastrocnemius lateralis; RF, rectus femoris; VM, vastus medialis; VL, vastus lateralis; ST, semitendinosus; BF, biceps femoris; TFL, tensor fasciae latae; AL, adductor longus; GLMax, gluteus maximus; GLMed, gluteus medius; Uni, unilateral; Bi, bilateral; NMF, non-negative matrix factorization; NG, not given*.

### Quantification of Synergies

The quantification of synergies was often done *post-hoc* based on the variance of EMG activity accounted for (VAF). VAF is a measure of the quality of the EMG reconstruction based on the selected number of muscle synergies. Twelve of the 16 studies included in this review reported the total number of synergies during walking using a certain VAF threshold (Torricelli et al., 2014; Steele et al., 2015, 2019; Tang et al., 2015; Cappellini et al., 2016, 2018; Shuman et al., 2017, 2019; Hashiguchi et al., 2018; Kim Y. et al., 2018; Oudenhoven et al., 2019; Yu et al., 2019). Out of these articles, nine compared the number of synergies between individuals with CP and unimpaired individuals (Steele et al., 2015; Tang et al., 2015; Cappellini et al., 2016, 2018; Shuman et al., 2017, 2019; Hashiguchi et al., 2018; Kim Y. et al., 2018; Yu et al., 2019), while the remaining three only included children with CP in their study (Torricelli et al., 2014; Oudenhoven et al., 2019; Steele et al., 2019). Despite a difference in VAF threshold, number of subjects, and number of recorded muscles (see Table 3), the majority of studies found that children with CP recruited fewer synergies (range 1–4) compared to TD children (range 3–4) or healthy adults (all 4) on average when comparing the number of synergies during walking (Torricelli et al., 2014; Steele et al., 2015; Tang et al., 2015; Shuman et al., 2017; Hashiguchi et al., 2018; Kim Y. et al., 2018; Yu et al., 2019). In contrast, Cappellini et al. (2016, 2018) found that both children with CP and TD children recruited 4 synergies. They used a linear regression procedure that plots the VAF against the number of synergies and finds the smallest number for which the root mean square error of the corresponding linear fit is smaller than 10^−4^ (d'Avella et al., 2006). The authors show that this corresponds to a VAF>80% for all subjects.

Six studies reported VAF_1_, the variation of EMG activity that can be explained by just one synergy, which is another parameter computed to study the complexity of the locomotor behavior (Steele et al., 2015, 2019; Shuman et al., 2016, 2017; Goudriaan et al., 2018; Kim Y. et al., 2018). Five of the six studies found that the average VAF_1_ was significantly larger in children with CP (range 71.0–84.2%) compared to TD children (range 61.0–74.7%, see Table 1). Steele et al. (2019) did not compare with TD children, but showed that VAF_1_ was 81.4 ± 5.5% for children with CP.

Three studies reported the Dynamic Motor Control index during walking (Steele et al., 2015; Shuman et al., 2017; Kim Y. et al., 2018; walk-DMC), which is associated to VAF_1_, for comparisons of muscle synergies between children with CP and TD children. Walk-DMC transforms VAF_1_ to a z-score with respect to TD children. A score of 100 signifies the average walk-DMC of TD children and each 10-point interval is one standard deviation. Steele et al. (2015) proposed this measure as a clinical tool to quantify altered neuromuscular control, in order to plan treatments and predict clinical outcomes. In agreement with the results on VAF_1_, all three studies found significantly lower walk-DMC values in children with CP (range of averages 65.0–86.2) compared to TD children (average 100; Steele et al., 2015; Shuman et al., 2017; Kim Y. et al., 2018). One of these studies showed that an increase in low-pass filter cut-off frequency from 4 to 40 Hz caused an increase in the total number of synergies, and a decrease in VAF_1_ in both children with CP and TD children. However, it had no effect on walk-DMC, since this measure normalizes VAF_1_ to a z-score (Shuman et al., 2017).

### Structure of Synergies

Eight studies compared the structure of synergies in terms of the results on temporal and spatial patterns between children with CP and controls (Torricelli et al., 2014; Steele et al., 2015; Tang et al., 2015; Cappellini et al., 2016, 2018; Kim Y. et al., 2018; Shuman et al., 2019; Yu et al., 2019). Two studies found that the spatial structure of synergies of children with CP was different from healthy adults (“mature synergies;” Torricelli et al., 2014; Tang et al., 2015), as was assessed by Tang et al. (2015) using a model called synergy comprehensive assessment. In addition, Tang et al. (2015) showed that the spatial structure of synergies in children with CP was different from TD children, and that a large variation in synergy structure was present in the CP group. The majority of children with CP showed a combination of “mature synergies” and synergies specific to CP, however none of the affected legs in children with unilateral CP showed merely “mature synergies.”

Six studies found that the spatial structure of synergies in both children with CP and TD children was related to that of “mature synergies,” but that the temporal structure differed between children with CP and TD children (Steele et al., 2015; Cappellini et al., 2016, 2018; Kim Y. et al., 2018; Shuman et al., 2019; Yu et al., 2019). These studies found differences in the duration and shifts of the peaks of the temporal patterns within the gait cycle in children with CP compared to TD children. In addition, Yu et al. (2019) showed larger co-activation between synergies and higher variability of the temporal patterns within groups (GMFCS I and II), in children with CP compared to TD children.

### Between-Subject Variability

Five studies discussed the muscle synergy differences within the heterogenous CP group (see Table 4). The relation between the severity of CP and muscle synergies was examined comparing between different distribution of CP, i.e. uni- or bilateral (Steele et al., 2015; Tang et al., 2015), and levels of impairment of functional mobility, as represented by GMFCS scores and/or Gillette Functional Assessment Questionnaire (Novacheck et al., 2000) scores (Steele et al., 2015; Tang et al., 2015; Schwartz et al., 2016; Hashiguchi et al., 2018; Kim Y. et al., 2018; Yu et al., 2019).

**Table 4:** Severity of CP.

**References**	**Distribution of CP**		**Level of impairment of functional mobility**		
	**Total N**	**Walk-DMC**	**Total N**	**Walk-DMC**	**Structure**
Tang et al. (2015)	Uni: 4 both legs Bi: 2 or 3 per leg	-	GMFCS I: 2 = 50%; 3 = 25%; 4 = 25% GMFCS II: 2 = 50%; 3 = 17%; 4 = 33% GMFCS III: 2 = 25%; 3 = 75% GMFCS IV: 2 = 100%	-	-
Steele et al. (2015)	-	He: 89.2 (87.8–90.6) Di: 86.9 (85.9–87.9) Tri: 84.4 (82.5–86.3) Quad: 81.4 (80.0–82.8)	-	GMFCS I: 92.4 (91.1–93.7) GMFCS IV: 79.2 (77.5–80.9) FAQ = 10: 90.9 (89.2–92.6) FAQ <7: 80.0 (78.7–81.3)	Higher GMFCS level = more synergy structures that are specific to CP
Yu et al. (2019)	-	-	GMFCS I/II = 4; GMFCS III = 3	**-**	GMFCS I: DAM = 29.8; GMFCS II: DAM = 30.6; TD: DAM = 26.4
Hashiguchi et al. (2018)	-	-	No correlation with GMFCS levels: χ^2^ = 4.06, *p* = 0.40	**-**	-
Kim Y. et al. (2018)	-	-	No correlation with GMFCS levels	**-**	GMFCS level was correlated with normalized cluster number: *r* = 0.51, *p* = 0.01

*CP, cerebral palsy; N, number of synergies; Walk-DMC, dynamic motor control index during walking; Uni, unilateral; Bi, bilateral; He, hemiplegic; Di, diplegic; Tri, triplegic; Quad, quadriplegic; GMFCS, gross motor function classification system; DAM, deviation of activation matrix (to identify variability of activations patterns between subjects)*.

Children with CP that were bilaterally affected recruited fewer synergies, as identified by lower walk-DMC scores (Steele et al., 2015), a lower total number of synergies, and synergy structures more specific to the CP group (Tang et al., 2015). In addition, higher GMFCS levels in children with CP were related to lower walk-DMC scores (Steele et al., 2015; Schwartz et al., 2016) and a lower total number of synergies (Tang et al., 2015; Yu et al., 2019). In contrast, Hashiguchi et al. (2018) and Kim Y. et al. (2018) did not find a correlation between the total number of synergies and GMFCS level, although Hashiguchi et al. (2018) found that a higher level of spasticity in children with CP, as assessed by the modified Ashworth Scale, was correlated with a lower number of synergies. The temporal structure of synergies was shown to differ between the affected and less affected side of children with unilateral CP and children with bilateral CP (Cappellini et al., 2016), and higher synergy variability was found in children with higher GMFCS levels (Kim Y. et al., 2018; Yu et al., 2019).

### Within-Subject Variability

No systematic differences in number, and spatial or temporal structure of synergies were found between days (Shuman et al., 2016; Steele et al., 2019). However, muscle synergies were found to be variable between strides in both children with CP and TD children (Shuman et al., 2016; Kim Y. et al., 2018). Kim Y. et al. (2018) used a cluster analysis based on a combination of iterative *k-*means clustering and intraclass correlation coefficient analyses to identify stride-to-stride variability of muscle synergies (Kim et al., 2016). The authors found that children with CP had a higher normalized cluster number, meaning that they showed more distinct clusters across strides, although they recruited fewer synergies. Thus, children with CP had higher variability in spatial and temporal synergy structure between strides compared to TD children, for various VAF thresholds (see Table 5).

**Table 5:** Variability of synergies.

**References**	**Between strides**		**Between days**	
	**VAF_**1**_ (%)**	**Structure**	**VAF_**1**_ (%)**	**Structure**
Shuman et al. (2016)	**Mean std:** CP = 5.0% (range 2.5–7.5%); TD = 4.9% (range 3.7–6.5%) **Mean max difference:** CP = 19.1%; TD = 18.2%	**-**	**Day 1:** CP = 77.4 ± 5.3%; TD = 68.4 ± 5.0% **Day 2:** CP = 76.9 ± 4.8%; TD = 68.4 ± 4.7%	-
Steele et al. (2019)	**-**	-	**Average change day 1 to day 2:** 4.24 ± 3.09%	**CS (day 1 vs. day 2):** 2 syn: w = 0.89 ± 0.10; act = 0.93 ± 0.06 3 syn: w = 0.83 ± 0.11; act = 0.91 ± 0.06 4 syn: w = 0.90 ± 0.08; act = 0.92 ± 0.05
Kim Y. et al. (2018)	**-**	Normalized cluster number* for VAF >80%: CP = 0.38 ± 0.06; TD = 0.33 ± 0.04 >85%: CP = 0.37 ± 0.07; TD = 0.31 ± 0.05 >90%: CP = 0.41 ± 0.05; TD = 0.34 ± 0.08 >95%: CP = 0.39 ± 0.05; TD = 0.34 ± 0.02	**-**	**-**

*CP, cerebral palsy; TD, typically developing; VAF_1_, variance accounted for by one synergy (%); std, standard deviation; max, maximum; CS, cosine similarity; syn, synergies; w, weights; act, activations*.

### Treatment

Three studies investigated whether muscle synergy characteristics in children with CP before treatment are predictive of the effect of different treatments, including selective dorsal rhizotomy (SDR), single-event multilevel orthopedic surgery (SEMLS), single-level orthopedic surgery, botulinum toxin type A (BoNT-A) injection or conservative treatment (physical therapy). Higher walk-DMC values before treatment were associated with improved gait quality, as defined by the Gait Deviation Index and walking speed, after several treatments (Schwartz et al., 2016; Shuman et al., 2018). A higher total number of synergies before treatment was associated with an improved knee angle at initial contact and midstance after SDR, but not with an improvement of overall gait quality, as quantified by the Edinburgh visual gait score (Oudenhoven et al., 2019).

Shuman et al. (2019) investigated whether muscle synergies change after treatment, and whether these changes were associated with treatment outcomes. They found no changes in the number of synergies, or synergy weights, and only minimal changes in VAF_1_ after BoNT-A and SDR. Temporal structure of synergies changed only after SDR, toward being more different from TD children. Children with CP whose synergies had a temporal structure more similar to TD children after treatment showed improved gait quality.

## Discussion

Walking problems in children with CP can in part be explained by limited selective motor control, i.e., the impaired ability to use the correct muscle group to move a joint independently from other joints in a limb during movement (Desloovere et al., 2006). Muscle synergy analysis is increasingly used to quantify altered neuromuscular control during walking. This systematic review analyzed 16 studies investigating muscle synergies in children with CP during walking, and aimed to examine how these synergies differ from those exhibited by TD children.

### Quantification of Synergies

The majority of studies found that children with CP recruit fewer synergies during walking compared to TD children, either based on a certain VAF threshold, VAF_1_, or walk-DMC (Torricelli et al., 2014; Steele et al., 2015, 2019; Tang et al., 2015; Schwartz et al., 2016; Shuman et al., 2016, 2017, 2018, 2019; Goudriaan et al., 2018; Hashiguchi et al., 2018; Kim Y. et al., 2018; Yu et al., 2019). The authors of these studies suggest that neuromotor control is altered or less complex in children with CP. The number of synergies for children with CP and TD children varied between studies. Cappellini et al. were the only ones that did not find a difference in terms of number of synergies between children with CP and TD children (Cappellini et al., 2016, 2018).

The differences in findings between studies may be a consequence of the varying functional mobility levels of subjects included by the different studies. Cappellini et al. (2016, 2018) included children with CP with a relatively high functional mobility level (77–79% GMFCS I) compared to the other studies (range 22–67% GMFCS I), with the exception of Shuman et al. (2016) (100% GMFCS I). It is plausible that the functional mobility of children with CP and TD children was too similar in Cappellini et al. (2016, 2018) to find a difference in the number of synergies between groups.

The use of different methods to define the total number of synergies may also impact synergy outcomes between studies (Hug et al., 2012; Russo et al., 2014). Most studies in this review used VAF to define the total number of synergies, several of these defined a specific VAF threshold, but there is no agreement on the optimal height of this threshold. Consequently, the VAF thresholds ranged from 80% to 95% across studies, affecting the total number of synergies that are considered. However, this only influences comparisons of the number of synergies between studies, but differences between groups within one study can still be observed. No systematic differences in number of synergies were found between studies in this review, using different VAF thresholds. To avoid the impact of this threshold, some studies used VAF_1_ or the related measure walk-DMC, and found results comparable to the VAF threshold. Cappellini et al. (2016, 2018) were the only ones using a different method to define the number of synergies, namely the “best linear fit” method. However, it is unlikely that the use of this method explains the similarity in total number of synergies between children with CP and TD children found in Cappellini et al. (2016, 2018), since the authors verified that their results agreed with a VAF > 80%. None of the studies in this review considered the added variance of the following synergy as a measure to define the total number of synergies (Clark et al., 2010). The added variance could be an extra tool in the future to define the total number of synergies as it negates the risk that a synergy does not contribute sufficiently to the muscle activation pattern of interest.

The variation in synergy outcomes between studies could also be explained by the different number of muscles recorded. According to previous research, a low number of muscles used for analysis could lead to an over-estimation of VAF (Steele et al., 2013; Zelik et al., 2014; Damiano, 2015). Several studies used NMF to decompose four to eight muscles into two to four synergies, but it is debatable whether this reduction aids enough in terms of easing the interpretation of the data from a statistical point of view. Yet, since it is not feasible to measure all muscles involved in walking, a decomposition will always approximate true neural signaling. Cappellini et al. (2016, 2018) were the only ones recording a large number of bilateral muscles, 11 per leg, which may result in a more precise estimation of the muscle synergies involved during walking. This could possibly explain in part why they did not find differences between CP and TD, while others did.

In addition, processing methods of the EMG data, such as filters and amplitude scaling, have been shown to influence muscle synergy outcomes (Shuman et al., 2017). The majority of studies included in this review used a low-pass filter with a cut-off frequency of 10 Hz, but some studies used low-pass filters of 2 Hz (Oudenhoven et al., 2019), 4 Hz (Shuman et al., 2016), and 5 Hz (Torricelli et al., 2014; Kim Y. et al., 2018). The lower the low-pass cut-off frequency, the more data is attenuated, which has been shown to result in a lower number of synergies (van der Krogt et al., 2016; Shuman et al., 2017), and smaller increases in VAF post-treatment (van der Krogt et al., 2016). There is no consensus yet on the best cut-off frequency for a low-pass filter. Different filter types and filter orders are used across studies, but these choices appear to be less significant than the low-pass cut-off frequency (Devaprakash et al., 2016). The influence of methodological choices on muscle synergies is especially important to consider when comparing results across studies or between centers, using different ways to process their data. Overall, despite differences in the number and choice of muscles, and EMG preprocessing methods, studies found similar results. Moreover, the methods were the same in the CP and TD group within all studies and should therefore have an equal effect on the muscle synergies of all groups. Consequently, these factors are not likely to explain the lack of difference in number of synergies between children with CP and TD children found by Cappellini et al. (2016, 2018).

### Structure of Synergies

A subset of the included studies examined differences in the structure of muscle synergies between children with CP and TD children (Torricelli et al., 2014; Steele et al., 2015; Tang et al., 2015; Cappellini et al., 2016, 2018), but they showed different results. Some studies found differences in the spatial structure, i.e., muscle weights, between children with CP and TD children (Torricelli et al., 2014; Tang et al., 2015), whereas others only found differences in the temporal structure, i.e. timing and duration of the peaks of the temporal activation patterns (Torricelli et al., 2014; Steele et al., 2015; Cappellini et al., 2016, 2018; Kim Y. et al., 2018; Yu et al., 2019).

Variation in the use of amplitude scaling methods could result in a different weighting of the synergies per muscle. Scaling to unit variance appears to reduce these differences in muscle weights, with more consistent synergy structures across low-pass filters and at a lower number of calculated synergies compared to peak amplitude scaling (Shuman et al., 2017). Although the differences were small, this finding might be specifically interesting for research investigating muscle synergies in clinical populations, which recruit fewer synergies compared to TD children. Moreover, normalization to individual maxima could distort the relative muscle weights due to variable weakness in CP, which can result in inconsistent findings on the spatial structure of synergies across studies (Damiano, 2015).

Deviation from the structure of “mature synergies” in children with CP was found (Torricelli et al., 2014; Tang et al., 2015), and could be a result of the lack of fractionation of synergies, i.e., splitting of one synergy into more, during development. Previous research in stroke patients suggests that a lower number of synergies could result from merging of the synergies of healthy controls (Clark et al., 2010; Cheung et al., 2012). Merging and fractionation of synergies influenced the longitudinal changes of walking patterns in patients after subacute stroke, whereas the number of synergies did not (Cheung et al., 2012; Hashiguchi et al., 2016). Therefore, it might add value to examine the structure including possible fractionation of synergies.

The studies used different methods to quantify similarity between synergy structure. Torricelli et al. (2014) compared the temporal activation patterns using adult data (Winter, 1991), not specifying the method they used, while Tang et al. (2015) and Yu et al. (2019) used Pearson's correlation coefficients, and the other studies used cluster analyses to compare the structure between subjects (Steele et al., 2015; Cappellini et al., 2016, 2018; Kim Y. et al., 2018). These cluster analyses identified comparable patterns across subjects. Three studies isolated the synergies that where not consistent across children as “Not Classified” (Steele et al., 2015; Cappellini et al., 2016, 2018). This means that the synergies that were specific to one child were not considered, and the authors did not quantify how many synergies were removed from each subject. Consequently, differences in synergies within the group of children with CP, and between children with CP and TD children were possibly lost, which could be a reason why these studies did not find (large) differences in synergy structure between children with CP and TD children. Kim Y. et al. (2018) did allow synergy structures to be assigned to more clusters, and they also found similar synergy structures between children with CP and TD children. However, children with CP recruited fewer synergies per stride, and the use of these structures was less consistent across strides. This means that relative to the number of synergies per stride, children with CP could access more synergy structures than TD children, which suggests that children with CP exhibit the same complexity of synergy structures, but the control of these structures might be decreased. In order to confirm this idea, more studies using the same clustering method are necessary.

Cappellini et al. (2016) found similarities in temporal structure of synergies between children with CP and TD toddlers (1–1.2 years of age) who just started to walk independently. This suggests that muscle synergies in children with CP lag behind in development compared to TD children, which agrees with previous research showing similarity between the walking pattern in children with CP and early gait in TD children (Berger et al., 1982, 1984; Leonard et al., 1991).

### Variability of Synergies

The variation in findings between studies on the number and structure of synergies might be related to the differences in distribution and levels of functional mobility in CP. Children with more severe types of CP, defined by either more distributed CP or higher GMFCS levels, were found to use fewer synergies (Steele et al., 2015; Tang et al., 2015), with different spatial (Tang et al., 2015) and temporal (Steele et al., 2015; Cappellini et al., 2016; Shuman et al., 2019) structures compared to less affected children. These results might reflect a simpler motor control strategy during walking with increasing severity of CP.

In contrast, Hashiguchi et al. (2018) and Kim Y. et al. (2018) did not find a relationship between number of synergies and GMFCS level, possibly because of the small sample size, which limits the variability in a group. Tang et al. (2015) and Yu et al. (2019) also included a limited group of children and they did find an effect of GMFCS level on the number of synergies. Thus, the relationship between the severity of CP and muscle synergies is shown in studies with a sufficient number of subjects (Steele et al., 2015; Cappellini et al., 2016; Shuman et al., 2019), but small sample sizes can coincidentally not show it.

One study found a higher stride-to-stride variability in muscle synergies in children with CP (Kim Y. et al., 2018). This may represent a more immature walking pattern (Hausdorff et al., 1999). High stride-to-stride variability can influence VAF values and thus impact the decomposition of the data into muscle synergies. Only four studies used the minimum of about 20 strides that is necessary according to Oliveira et al. (2014) to create optimal reconstructions of the data and minimize the influence of the variability between strides (Tang et al., 2015; Cappellini et al., 2016, 2018; Shuman et al., 2016). Based on the low amount of studies in this review assessing specifically this aspect we cannot infer whether a lower number of analyzed strides could have an effect on a lower number of synergies.

Considering the high diversity within the group of children with CP, it is not surprising that many studies found larger variability in number and structure of muscle synergies in children with CP compared to TD children. In some studies children with more severe types of CP walked with an assistive device or trunk or hand support (Tang et al., 2015; Hashiguchi et al., 2018; Kim Y. et al., 2018; Oudenhoven et al., 2019; Shuman et al., 2019; Yu et al., 2019). In addition, children with more severe types of CP generally walk slower compared to less affected and TD children. Walking speed is an important factor to consider when evaluating muscle synergies, as previous research found that both number and structure of synergies were affected by walking speed in healthy adults (Yokoyama et al., 2016; Kibushi et al., 2018) and TD children (Steele et al., 2015). These findings suggest that different walking speeds require different control from the central nervous system. However, others found that muscle synergies were robust across different walking speeds in healthy adults (Ivanenko et al., 2004; Chvatal and Ting, 2012) and children with CP (Tang et al., 2015; Hashiguchi et al., 2018). Although findings are inconsistent, walking speed as a possible confounding factor in comparisons of muscle synergies between children with CP and TD children should be considered during muscle synergy analysis. In addition, the quality of EMG data and the absence of task-independent normalization may have caused variation in muscle synergy results between studies, and should be considered in the future.

### Treatment

The finding that muscle synergies before treatment were correlated with the effect of treatment in children with CP (Shuman et al., 2016, 2018; Oudenhoven et al., 2019), suggests that knowledge about muscle synergies in children with CP before treatment could help predict whether children will benefit from a specific treatment, and therefore potentially assist in treatment decisions. Walk-DMC has been proposed as a possible measure to quantify altered neuromuscular control pre-treatment, since it has been shown to be correlated with improvement of gait kinematics and walking speed after treatment (Schwartz et al., 2016; Shuman et al., 2018). Importantly, EMG processing methods, and number and type of muscles have limited impact on walk-DMC values. Therefore, this measure could be useful as a comparison of muscle synergy analyses across studies or different clinical centers using different EMG protocols. However, walk-DMC values are highly variable in a heterogeneous population like CP (Steele et al., 2015; Shuman et al., 2018). Although the mean results of walk-DMC values using a large sample size might be a good predictor of treatment outcome, caution should be taken when using individual walk-DMC values in treatment prediction.

Besides the use of muscle synergies as a predictor of treatment outcomes, muscle synergies may also be a target for treatment themselves. Younger children with CP might be more sensitive to interventions (Yang et al., 2013), because their brain is highly plastic and their corticospinal tract is still maturing. Future research should examine the opportunities of specific therapies that target the neural level and adapt muscle synergies, to improve the walking pattern of children with CP. Previous research in unimpaired individuals showed that both the spatial and temporal structure of muscle synergies can change due to intense training in elite athletes (Sawers et al., 2015; Kim M. et al., 2018), and with the use of ankle exoskeletons (Steele et al., 2017; Jacobs et al., 2018). However, current treatments studied in CP were found to have no effect on the spatial structure and merely an effect on the temporal structure of muscle synergies (Shuman et al., 2019). These results suggest that the number and spatial structure of synergies may be hard to change in children with CP, but that the temporal structure of synergies could be a target for treatment. However, normalization of the EMG data is an important factor that may have influenced the results on the spatial structure of synergies. It remains to be further investigated whether novel treatments, such as feedback training (Booth et al., 2019), or therapeutic electrical stimulation of muscles, tendons (Sommerfelt et al., 2001; í et al., 2002; Stackhouse et al., 2007; Wright et al., 2012), or spinal cord (Solopova et al., 2017), could improve muscle synergies, eventually leading to walking improvement.

### Future Directions

The number of studies currently available on this topic is limited, which makes it difficult to draw additional conclusions. With this systematic review we hope to inform researchers about the current research status and to guide them toward better research in the future.

The large variation in number and structure of muscle synergies derived from children with CP appears to reflect the diversity of CP and the ability of walking. However, methodological factors also seem to play a role in the determination of muscle synergies. On the one hand, it will be helpful when studies investigating muscle synergies in children with CP use consistent methods across different studies, in order to compare results. On the other hand, this would limit researchers to explore and use novel technologies. At least, researchers could consider recording a number of muscles that is representative for the muscle activation during walking, as well as a sufficient number of strides, in order to make a proper decomposition of muscle synergies. To achieve consistency in EMG data processing steps across studies, researchers should be informed about the choice of filters and factorization methods. The determination of a suitable method to process EMG data of children with CP during walking, for example with a standard EMG processing pipeline, is an important area for future research. If the group of children with CP is heterogeneous, muscle synergy analysis should be performed on separate groups based for example on different distribution of CP (i.e., uni- or bilateral CP) or different functional mobility levels with sufficient sample sizes, in order to examine the diversity in the CP group. In addition, study of the influence of walking speed on muscle synergies in children with CP and TD children could be useful in the interpretation of the results found in the studies included in this review. Irrespective of the differences in data collection and analysis, the majority of the studies included in this review found similar results, which indicates that the difference in muscle synergies between CP and TD we observe is robust. These corresponding findings from different studies and research groups, provide strong evidence that the observations are related to neural control, and do not merely reflect methodological choices.

It is worth to mention that all the studies reported in this review used the so-called synchronous synergy model (time-invariant synergy approach) to investigate muscle synergies during walking in children with CP. However, various other models such as the time-varying synergy model, first introduced by d'Avella and Tresch (2002), or the space-by-time model (Delis et al., 2014) exist, and could be implemented to study muscle activation modularity in children with CP.

Investigation of the longitudinal development of muscle synergies within subjects would minimize the inter-subject variability and give more insight in the developmental changes in children with CP. Moreover, nothing is known about the development of muscle synergies in very young children at high risk of CP compared to TD children. A longitudinal design with consecutive measurements within subjects could give new insights in the development of muscle synergies during walking in children with CP, and might open up new paradigms for early interventions in CP.

Despite the increasing number of studies investigating muscle synergies, the underlying mechanisms of muscle synergies remain unknown. It is still a topic of debate whether muscle synergies have a neural or non-neural origin (Bizzi and Cheung, 2013; Zandvoort et al., 2019). Muscle synergies in neonates were shown to mainly reflect spinal cord and brainstem activity, with an increase of the integration of supraspinal and sensory control during development (Dominici et al., 2011). Even though children with CP have cortical lesions, the differences in muscle synergies compared to TD children might also depend on changes in the brainstem and/or spinal cord. In addition, it is debatable whether the use of fewer muscle synergies necessarily reflects less complex motor control, as is suggested in most studies, or whether it is merely caused by higher variability in the EMG data in children with CP. Further research on the underlying mechanisms of muscle synergies is required to answer these questions.

## Conclusions

In conclusion, the majority of studies found that children with CP recruit fewer synergies than TD children, and differences in both spatial and temporal structure of synergies were found. In addition, large variability of muscle synergies was found in the group of children with CP, which might be due to the heterogeneity in this group with different functional mobility levels of CP. The inter-subject variability in number and structure of synergies was higher in children with more severe CP, and within subjects the stride-to-stride variability was higher in children with CP compared to TD children, which is known to influence VAF values and thus impact the decomposition of the EMG data into muscle synergies.

The findings in this systematic review support the idea that children with CP use a simpler motor control strategy compared to TD children. The use of muscle synergies as a clinical tool to quantify altered neuromuscular control and predict clinical outcomes seems promising. Further investigation on this topic is necessary, and the use of muscle synergies as a target for development of novel therapies in children with CP could be explored.

## Data Availability Statement

The raw data supporting the conclusions of this article will be made available by the authors, without undue reservation, to any qualified researcher.

## Author Contributions

AB and MB performed the search, quality assessment and data analysis. RV contributed to the search of databases. AB carried out the drafting of the manuscript. All authors contributed to the manuscript revisions, and approved the submitted version.

## Conflict of Interest

The authors declare that the research was conducted in the absence of any commercial or financial relationships that could be construed as a potential conflict of interest.

## Abbreviations

BoNT-A: Botulinum Toxin Type A
CP: Cerebral Palsy
EMG: Electromyography
GMFCS: Gross Motor Function Classification System
NMF: Non-Negative Matrix Factorization
SDR: Selective Dorsal Rhizotomy
SEMLS: Single-Event Multilevel Surgery
TD: Typically Developing
VAF: Variance Accounted For
VAF_1_: Variance Accounted For by one synergy
Walk-DMC: Dynamic Motor Control index during walking.
