# Supplementary material for: Muscle Synergies During Walking in Children With Cerebral Palsy: A Systematic Review
Source: Front Physiol. 2020 Jul 2;11:632. doi: 10.3389/fphys.2020.00632 (PMC7343959; doi:10.3389/fphys.2020.00632)
Supplement: Supplementary file 1 [file Table_1.DOCX]

Supplementary Material

# Appendix

**PubMed Session Results (22 Apr 2019)**

| Search | Query | Items found |
| --- | --- | --- |
| [#11](https://www.ncbi.nlm.nih.gov/pubmed) | #8 OR #10 | [2](https://www.ncbi.nlm.nih.gov/pubmed/?cmd=HistorySearch&querykey=11)93 |
| [#10](https://www.ncbi.nlm.nih.gov/pubmed) | #3 AND #9 | [2](https://www.ncbi.nlm.nih.gov/pubmed/?cmd=HistorySearch&querykey=10)59 |
| [#9](https://www.ncbi.nlm.nih.gov/pubmed) | child*[tw] OR schoolchild*[tw] OR preschool*[tw] OR infan*[tw] OR pediatri*[tw] OR paediatr*[tw] OR boy[tw] OR boys[tw] OR boyhood[tw] OR girl[tw] OR girls[tw] OR girlhood[tw] OR youth*[tw] OR preadolescent*[tw] OR adolescent*[tw] OR youngster*[tw] OR toddler*[tw] OR newborn*[tw] OR neonat*[tw] OR baby[tw] OR baby's[tw] OR babies[tw] | [4,107,792](https://www.ncbi.nlm.nih.gov/pubmed/?cmd=HistorySearch&querykey=9) |
| [#8](https://www.ncbi.nlm.nih.gov/pubmed) | #5 OR #7 | [137](https://www.ncbi.nlm.nih.gov/pubmed/?cmd=HistorySearch&querykey=8) |
| [#7](https://www.ncbi.nlm.nih.gov/pubmed) | #3 AND #6 | 3[2](https://www.ncbi.nlm.nih.gov/pubmed/?cmd=HistorySearch&querykey=7) |
| [#6](https://www.ncbi.nlm.nih.gov/pubmed) | typically develop*[tiab] OR typical child*[tiab] OR unimpaired child*[tiab] OR healthy child*[tiab] | [31,](https://www.ncbi.nlm.nih.gov/pubmed/?cmd=HistorySearch&querykey=6)773 |
| [#5](https://www.ncbi.nlm.nih.gov/pubmed) | #3 AND #4 | [1](https://www.ncbi.nlm.nih.gov/pubmed/?cmd=HistorySearch&querykey=5)26 |
| [#4](https://www.ncbi.nlm.nih.gov/pubmed) | "Cerebral Palsy"[Mesh] OR cerebral pals*[tiab] OR brain pals*[tiab] OR brain paralys*[tiab] OR central pals*[tiab] OR central paralys*[tiab] OR cerebral paralys*[tiab] OR cerebral pares*[tiab] OR (encephalopathi*[tiab] AND infantil*[tiab]) OR spastic*[tiab] OR little disease*[tiab] OR little's disease*[tiab] OR CP child*[tiab] | [51,](https://www.ncbi.nlm.nih.gov/pubmed/?cmd=HistorySearch&querykey=4)513 |
| [#3](https://www.ncbi.nlm.nih.gov/pubmed) | #1 AND #2 | [1,](https://www.ncbi.nlm.nih.gov/pubmed/?cmd=HistorySearch&querykey=3)959 |
| [#2](https://www.ncbi.nlm.nih.gov/pubmed) | (synerg*[tiab] AND (muscle*[tiab] OR muscular[tiab])) OR activation pattern*[tiab] OR non-negative matrix factori*[tiab] OR nonnegative matrix factori*[tiab] OR activation component*[tiab] OR activation timing*[tiab] OR motor program*[tiab] OR motor pattern*[tiab] OR selective motor control*[tiab] OR dynamic motor control*[tiab] OR common drive[tiab] OR (factor analys*[tiab] AND (muscle*[tiab] OR muscular[tiab])) OR (principal component analys*[tiab] AND (muscle*[tiab] OR muscular[tiab])) | [19,](https://www.ncbi.nlm.nih.gov/pubmed/?cmd=HistorySearch&querykey=2)480 |
| [#1](https://www.ncbi.nlm.nih.gov/pubmed) | "Locomotion"[Mesh:NoExp] OR "Running"[Mesh] OR "Walking"[Mesh] OR locomotion[tiab] OR running[tiab] OR gait[tiab] OR gaits[tiab] OR walk[tiab] OR walking[tiab] OR stepping[tiab] OR crawling[tiab] OR ambulati*[tiab] | [235,](https://www.ncbi.nlm.nih.gov/pubmed/?cmd=HistorySearch&querykey=1)608 |

[**Embase.com**](http://embase.com/)**Session Results (22 Apr 2019)**

| Search | Query | Items found |
| --- | --- | --- |
| [#11](https://www.ncbi.nlm.nih.gov/pubmed) | #8 OR #10 | 440 |
| [#10](https://www.ncbi.nlm.nih.gov/pubmed) | #3 AND #9 | 368 |
| [#9](https://www.ncbi.nlm.nih.gov/pubmed) | 'child'/exp OR 'adolescent'/exp OR 'adolescence'/exp OR 'childhood'/exp OR 'newborn period'/exp OR 'perinatal period'/exp OR 'pediatrics'/exp  OR child*:ab,ti,kw OR schoolchild*:ab,ti,kw OR preschool*:ab,ti,kw OR infan*:ab,ti,kw OR pediatri*:ab,ti,kw OR paediatr*:ab,ti,kw OR boy:ab,ti,kw OR boys:ab,ti,kw OR boyhood:ab,ti,kw OR girl:ab,ti,kw OR girls:ab,ti,kw OR girlhood:ab,ti,kw OR youth*:ab,ti,kw OR preadolescent*:ab,ti,kw OR adolescent*:ab,ti,kw OR youngster*:ab,ti,kw OR toddler*:ab,ti,kw OR newborn*:ab,ti,kw OR neonat*:ab,ti,kw OR baby:ab,ti,kw OR 'baby s':ab,ti,kw OR babies:ab,ti,kw | [4,349,](https://www.ncbi.nlm.nih.gov/pubmed/?cmd=HistorySearch&querykey=9)317 |
| [#8](https://www.ncbi.nlm.nih.gov/pubmed) | #5 OR #7 | 239 |
| [#7](https://www.ncbi.nlm.nih.gov/pubmed) | #3 AND #6 | 47 |
| [#6](https://www.ncbi.nlm.nih.gov/pubmed) | 'typically develop*':ab,ti,kw OR 'typical child*':ab,ti,kw OR 'unimpaired child*':ab,ti,kw OR 'healthy child*':ab,ti,kw | 43,448 |
| [#5](https://www.ncbi.nlm.nih.gov/pubmed) | #3 AND #4 | 222 |
| [#4](https://www.ncbi.nlm.nih.gov/pubmed) | 'cerebral palsy'/exp OR 'cerebral pals*':ab,ti,kw OR 'brain pals*':ab,ti,kw OR 'brain paralys*':ab,ti,kw OR 'central pals*':ab,ti,kw OR 'central paralys*':ab,ti,kw OR 'cerebral paralys*':ab,ti,kw OR 'cerebral pares*':ab,ti,kw OR (encephalopathi*:ab,ti,kw AND infantil*:ab,ti,kw) OR spastic*:ab,ti,kw OR 'little disease*':ab,ti,kw OR 'little s disease*':ab,ti,kw OR 'cp child*':ab,ti,kw | 67,945 |
| [#3](https://www.ncbi.nlm.nih.gov/pubmed) | #1 AND #2 | 2,680 |
| [#2](https://www.ncbi.nlm.nih.gov/pubmed) | 'muscle synergy'/exp OR (synerg*:ab,ti,kw AND (muscle*:ab,ti,kw OR muscular:ab,ti,kw)) OR 'activation pattern*':ab,ti,kw OR 'non-negative matrix factori*':ab,ti,kw OR 'nonnegative matrix factori*':ab,ti,kw OR 'activation component*':ab,ti,kw OR 'activation timing*':ab,ti,kw OR 'motor program*':ab,ti,kw OR 'motor pattern*':ab,ti,kw OR 'selective motor control*':ab,ti,kw OR 'dynamic motor control*':ab,ti,kw OR 'common drive':ab,ti,kw OR ('factor analys*':ab,ti,kw AND (muscle*:ab,ti,kw OR muscular:ab,ti,kw)) OR ('principal component analys*':ab,ti,kw AND (muscle*:ab,ti,kw OR muscular:ab,ti,kw)) | 24,919 |
| [#1](https://www.ncbi.nlm.nih.gov/pubmed) | 'locomotion'/de OR 'walking'/exp OR 'running'/exp OR locomotion:ab,ti,kw OR running:ab,ti,kw OR gait:ab,ti,kw OR gaits:ab,ti,kw OR walk:ab,ti,kw OR walking:ab,ti,kw OR stepping:ab,ti,kw OR crawling:ab,ti,kw OR ambulati*:ab,ti,kw | 346,653 |

**Web of Science Session Results (22 Apr 2019)**

| Search | Query | Items found |
| --- | --- | --- |
| [#11](https://www.ncbi.nlm.nih.gov/pubmed) | #8 OR #10 | 394 |
| [#10](https://www.ncbi.nlm.nih.gov/pubmed) | #3 AND #9 | 324 |
| [#9](https://www.ncbi.nlm.nih.gov/pubmed) | TS=("child*" OR "schoolchild*" OR "preschool*" OR "infan*" OR "pediatri*" OR "paediatr*" OR "boy" OR "boys" OR "boyhood" OR "girl" OR "girls" OR "girlhood" OR "youth*" OR "preadolescent*" OR "adolescent*" OR "youngster*" OR "toddler*" OR "newborn*" OR "neonat*" OR "baby" OR "baby's" OR "babies") | [2](https://www.ncbi.nlm.nih.gov/pubmed/?cmd=HistorySearch&querykey=9),654,642 |
| [#8](https://www.ncbi.nlm.nih.gov/pubmed) | #5 OR #7 | 208 |
| [#7](https://www.ncbi.nlm.nih.gov/pubmed) | #3 AND #6 | 36 |
| [#6](https://www.ncbi.nlm.nih.gov/pubmed) | TS=("typically develop*" OR "typical child*" OR "unimpaired child*" OR "healthy child*") | 31,908 |
| [#5](https://www.ncbi.nlm.nih.gov/pubmed) | #3 AND #4 | 196 |
| [#4](https://www.ncbi.nlm.nih.gov/pubmed) | TS=("cerebral pals*" OR "brain pals*" OR "brain paralys*" OR "central pals*" OR "central paralys*" OR "cerebral paralys*" OR "cerebral pares*" OR ("encephalopathi*" AND" infantil*") OR "spastic*" OR "little disease*" OR "little’s disease*" OR "CP child*") | 47,440 |
| [#3](https://www.ncbi.nlm.nih.gov/pubmed) | #1 AND #2 | 2,792 |
| [#2](https://www.ncbi.nlm.nih.gov/pubmed) | TS=(("synerg*" AND ("muscle*" OR "muscular")) OR "activation pattern*" OR "non-negative matrix factori*" OR "nonnegative matrix factori*" OR "activation component*" OR "activation timing*" OR "motor program*" OR "motor pattern*" OR "selective motor control*" OR "dynamic motor control*" OR "common drive" OR ("factor analys*" AND ("muscle*" OR "muscular")) OR ("principal component analys*" AND ("muscle*" OR "muscular"))) | 25,670 |
| [#1](https://www.ncbi.nlm.nih.gov/pubmed) | TS=("locomotion" OR "running" OR "gait" OR "gaits" OR "walk" OR "walking" OR "stepping" OR "crawling" OR "ambulati*") | 338,500 |
